# Is Wild Marine Biota Affected by Microplastics?

**DOI:** 10.3390/ani13010147

**Published:** 2022-12-30

**Authors:** Nunziatina Porcino, Teresa Bottari, Monique Mancuso

**Affiliations:** 1 Institute for Marine Biological Resources and Biotechnology (IRBIM)—CNR, 98122 Messina, Italy; 2 Department of Integrative Marine Ecology, Stazione Zoologica “Anton Dohrn”, Sicily Marine Centre, 98167 Messina, Italy

**Keywords:** microplastics, pollution, effects, marine biota

## Abstract

**Simple Summary:**

Microplastics are ubiquitous particles with dimensions less than 5 mm. In the marine environment, due to their small size, they can be ingested by organisms. The purpose of this review is to describe the negative effects related to the ingestion of microplastics in wild marine organisms. At the moment, few effects caused by the ingestion of microplastics in wild marine organisms are known.

**Abstract:**

The present review provides detailed information on the adverse effects of MPs on wild marine organisms, including tissue damage, fish condition, oxidative stress, immune toxicity, and genotoxicity. A bibliometric analysis was carried out on CiteSpace (version 6.1.R3) (Drexel University, Philadelphia, PA, USA) to verify how many papers studied the effects on wild marine species. The results showed a total of 395 articles, but only 22 really presented data on the effects or impacts on marine biota, and of these, only 12 articles highlighted negative effects. This review shows that the observed effects in wild organisms were less severe and milder than those found in the experimental conditions. The knowledge of negative effects caused by direct ingestion of microplastics in wild animals is still limited; more efforts are necessary to fully understand the role of MPs and the adverse effects on wild marine organisms, the ecosystem, and human health.

## 1. Introduction

The increasing amount of plastic pollution in the ocean is a global concern [1,2,3].

Global plastic production has a growing rate of about 5% per year, and in 2020, it was approximately 367 million tons [4]. About 10% of these new plastics are released into the marine environment [5]. The plastics that arrive in the sea are gradually degraded into ever smaller pieces up to microplastics (<5 mm) and nanoplastics (<1 μm) [6].

Recent studies documented the presence of microplastics (MPs) pollution worldwide, from the deep oceans to the polar areas [7,8,9,10,11]. MPs present in the marine environment can be ingested by a wide range of organisms, zooplankton, benthos, top predators, sea turtles, marine mammals, and birds [12,13,14,15,16].

One of the main scientific questions inferred in the last years refers to the potentially toxic effects of the MPs’ ingestion on the marine biota’s health status. MPs can induce toxic effects on organisms, affecting normal functioning, and may cause several organ-specific toxicities, such as neuronal, digestive, reproductive, and developmental toxicity [17,18,19,20,21,22,23]. Moreover, MPs may adsorb contaminants present in the environment because of their lipophilicity [24]; in fact, once arriving in the seawater, they change their nature in response to physicochemical and biological ageing processes [25] and, in this way, act as carriers for very hazardous chemicals (e.g., organic contaminants and heavy metals) [26,27]. MPs have sorb contaminants, such as heavy metals [28], pharmaceuticals [29], and persistent organic pollutants [30,31,32]. These pollutants can be ingested by marine organisms conveyed through MPs. Once ingested, the inflammatory cells and the detoxification mechanisms induce the generation of reactive oxygen species (ROS), which can produce oxidative stress and lipid peroxidation (LPO) of cellular membranes [33]. This oxidative stress can lead to an oxidative alteration of cellular components, including lipids, proteins, and DNA [34].

The organisms, to avoid oxidative stress and be protected from the toxic-induced damage, induce the activity of antioxidant enzymes such as catalase (CAT), superoxide dismutase (SOD), and glutathione peroxidase (GPx) [35]. Unfortunately, chronic exposure to MPs can cause oxidative damage [36]; for this reason, oxidative stress can be used as an indicator of physiological stress and can be measured indirectly by quantifying the antioxidant defenses and detoxification systems’ activity [34].

Finally, the fate of MPs once ingested by fish is not yet clearly understood [37,38]. MPs can reach internal tissues and organs such as the liver, the wall of the intestine, the stomach, and the muscle [39,40,41].

The presence of quantifiable levels of MPs in the muscle of edible fish species is worrying in regard to the potential consequences for human health [39]. In the last years, several studies on MPs’ toxicity were published [42]; however, knowledge of toxicological effects caused by direct consumption of microplastics in wild animals is still limited (Table 1).

The purpose of this review was to collect all the papers published up to date on the effects of microplastics on marine biota in order to have a global overview, but, above all, we aimed to understand what the real effects of microplastics on wild marine organisms are.

## 2. Materials and Methods

To carry out this bibliographic review, a list of references obtained from the Web of Science Database (WoS) (https://www.webofscience.com/wos/woscc/basic-search) (accessed on 23 November 2022) was used.

The searched keywords were “microplastics” AND “effects” AND “marine biota”. The titles, abstracts, and keywords were manually inspected to exclude irrelevant papers. The search included published original peer-reviewed research articles and reviews. The inclusion criteria applied for this review were as follows:

(1) Only the articles in the English language and published in peer-reviewed journals were considered in this paper; meanwhile, the technical reports, the monographs, the academic dissertations, the theses, and the conference proceedings were not included.
(2) Only articles that reported microplastic effects or impacts on the marine biota were included, while studies focused on sources in riverine or freshwater environments were not considered.
(3) Finally, articles that reported laboratory uptake experiments or modeling were not included in this review.

The analyses were performed with CiteSpace (version 6.1.R3), an open-source bibliometric software developed by Chen [62], and we analyzed 22 articles reporting the effects on marine biota. In this study, some parameters were considered to synthesize a stable network (1) term source (article title, abstract, and keywords), (2) node selection g-index (k = 50), (3) time slicing (years per slice) = 1, pruning (pathfinder and pruning sliced networks), and (4) visualization (cluster view-static and show merged network).

## 3. Results

### 3.1. Bibliometric Analysis 

**Figure 1:**
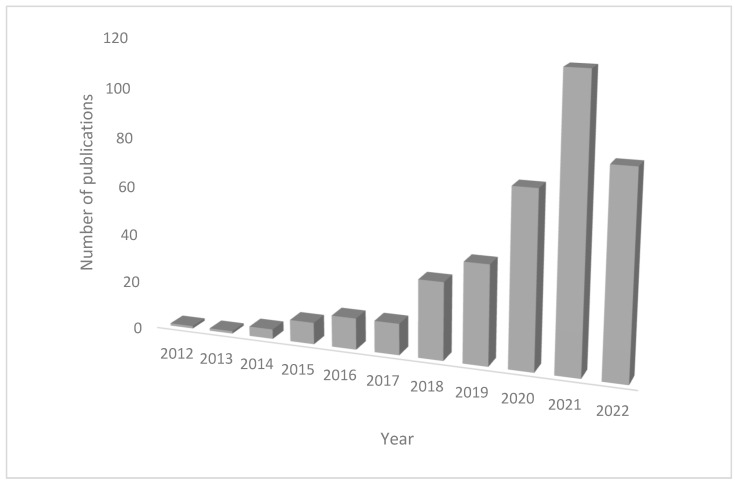
Number of publications relative to the research performed with WoS.

The results showed a total of 395 articles. By analyzing the results directly with WoS, it was possible to see the general impression of growing interest in MPs research (Figure 1).

Only 22 out of 395 articles really presented data on the adverse effects on wild marine biota. The first article was published in 2016 [52]. 

### 3.2. CiteSpace Analysis on MPs Effects in Wild Marine Organisms

Until now, the effects of the ingestion of MPs were evaluated in 27 marine species, including invertebrates, fish, birds, and reptiles, coming from the Northeast Atlantic Ocean, the Mediterranean Sea, and the Artic area (Table 1). 

#### 3.2.1. Country of Authorship and Affiliation

**Figure 2:**
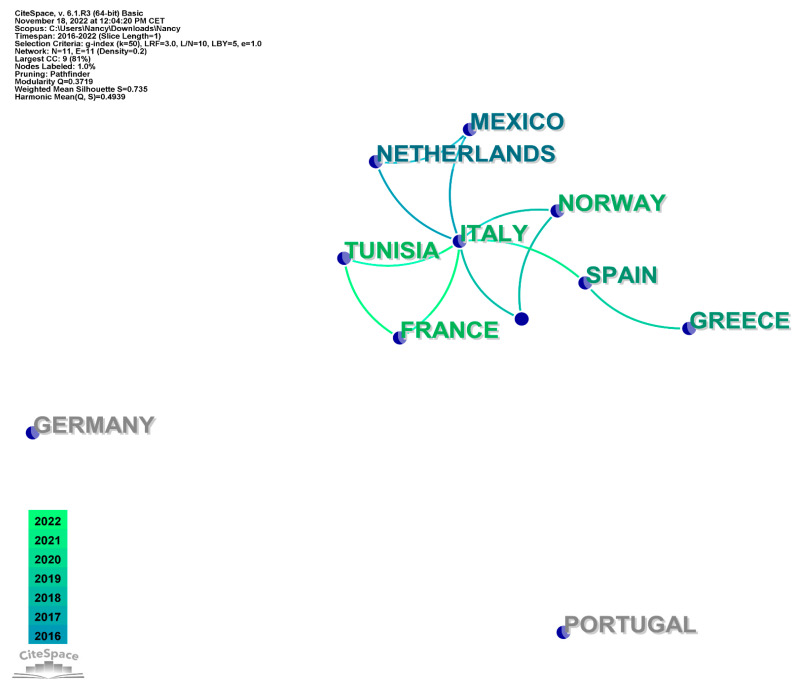
Distribution of the number of records attributed to authors based in each country.

The data analysis CiteSpace showed that the most represented authors belonged to Italy (35.5%), Spain (32.3%), Norway (6.5%), Greece, Portugal, Germany, Ireland, Tunisia, the Netherlands, France, and Mexico (3.2%) (Figure 2). 

**Figure 3:**
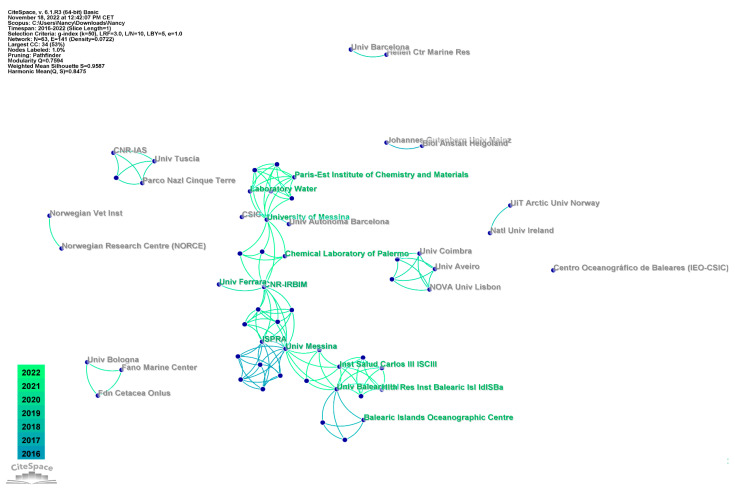
Distribution of organizations based on the number of records attributed to authors based in each organization.

The organizational analysis was used to reveal academic collaborations at the level of institutions (Figure 3).

The firsts ranked organizations with the largest research output were CNR-IRBIM (National Research Council)—Italy; University of Messina—Italy; University of the Balearic Islands—Spain; Istituto superiore per la protezione e la ricerca ambientale (ISPRA)—Italy; Instituto de Salud Carlos III—Spain; Balearic Islands Oceanographic Centre—Spain; Universidad Autonoma de Barcelona—Spain; University of Ferrara—Italy; and University of Siena—Italy.

#### 3.2.2. Keywords

**Figure 4:**
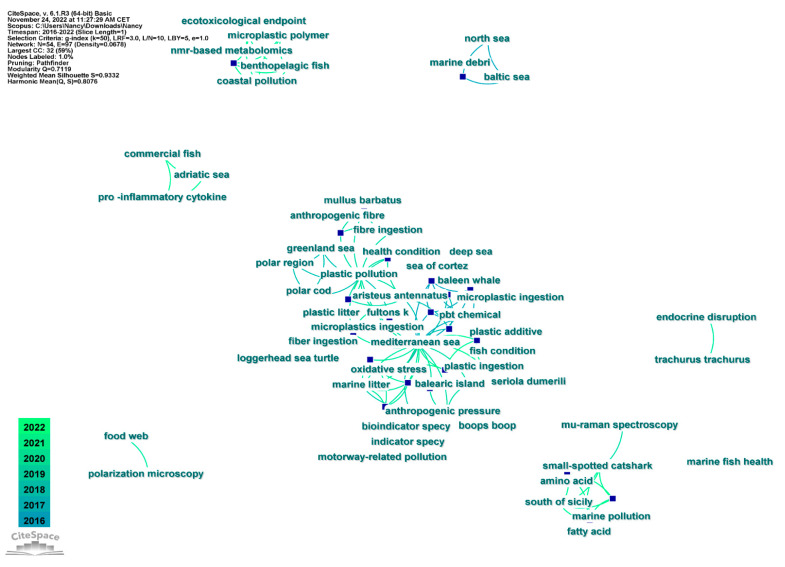
Keywords network, in which each node represents a keyword.

The keywords analysis showed that the first keywords of the articles related to our research were Mediterranean Sea (Citation Counts 10); plastic pollution (Citation Counts 5); oxidative stress, *Aristeus antennatus*, small-spotted catshark, marine litter, and Balearic Island (Citation Counts 2) (Figure 4).

#### 3.2.3. Journal Co-Citation Analysis

**Figure 5:**
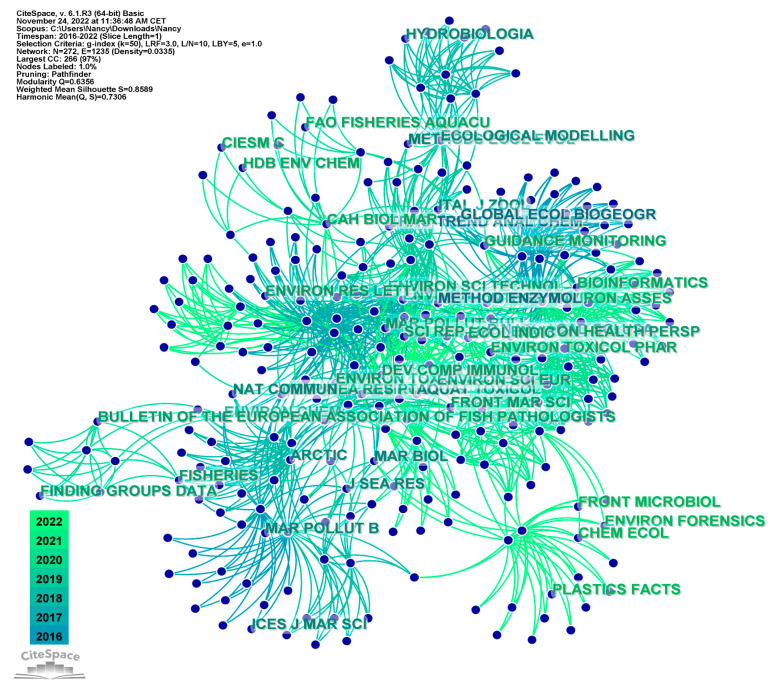
Cited journal networks.

The top-ranked items by citation counts were the *Marine Pollution Bulletin* and *Environmental Pollution*, with 19 counts; and Scientific Reports, *and Environmental Science & Technology*, with 18 counts. These journals are followed by *Marine Environmental Research*, *Science of the Total Environment*, and *Science*, with 16 citations; *Philosophical Transactions of the Royal Society B*, with 14 citations; and *Plos One*, with 13 citations. The 10th position is taken by *Environment International*, with 10 citations (Figure 5).

#### 3.2.4. Are There Any Gender Biases in the Authorship?

A study on the number of women in research was also carried out. In fact, generally, the women’s publication proportions are reduced with respect to the men, with the greatest discrepancy at the highest ranks [63]. The author’s position in a scientific article is very important; the most important positions are the first and the last. The first author is generally a scientist who performed most of the work, while the last author is the project leader or the supervisor.

Fifty-eight percent of the authors of the 22 articles that entered our database were women. Women represented 80% of all first authors in our database. The analysis of the latest authorships has also provided us with some preliminary conclusions regarding the academic condition of women in organizations dealing with this type of study, representing 75% of the latest authors (Table 2). Our data suggested to us that women studying the effects of microplastics on marine biota are well represented.

### 3.3. Effects of Microplastics

#### 3.3.1. Effects on the Brain (Neurotoxicity)

The brain is a tissue that is often used to determine the neurotoxic effects of environmental pollutants on marine organisms [34]. In this sense, neurotoxic effects were associated with the ingestion of MPs. Previous studies, under experimental conditions, evidenced the harmful effects of MPs ingestion in the brain of sea bass (*Dicentrarchus labrax*). MPs caused neurotoxicity through acetylcholinesterase (AChE) inhibition and increased lipid oxidation (LPO) [64].

The catalase (CAT), the superoxide dismutase (SOD), the glutathione-S-transferase (GST), the acetylcholine esterase (AchE), and the malondialdehyde (MDA) were measured in marine species that were naturally exposed to MPs’ ingestion (Table 1). Capó et al. [58] reported an increase in CAT activity (in *Mullus surmuletus* and *Boops boops*), in SOD activity (*Boops boops* and *Engraulis enchrasicolus*), and in GST activity (*Boops Boops*) as a consequence of MPs’ uptake. The AChE activity showed no differences related to MPs’ ingestion, as well as MDA levels (*M. surmuletus* and *B. boops*).

MPs’ contamination and effect biomarkers (brain acetylcholinesterase and lipid peroxidation) were evaluated by Barboza et al. [33] in wild *Dicentrarchus labrax, Trachurus trachurus*, and *Scomber colias*. The authors found that specimens that ingested MPs showed higher lipid peroxidation levels in the brain and increased brain acetylcholinesterase activity than specimens in which no MPs were found.

It is known that increased LPO levels indicate lipid peroxidation damage. The high concentrations of LPO in the brain could induce the break of acetylcholine-containing vesicles, resulting in increased neurotransmitters being released in the synaptic cleft [65]. Neurotoxicity by altered activity of acetylcholinesterase was also reported in *Serranus scriba* [43]. Finally, Hoyo-Alvarez et al. [34] reported that, in *Sparus aurata* specimens fed with MPs and a pollutants-enriched diet, the activity of some of the oxidative stress biomarkers increased; moreover, they showed the alterations in dopaminergic- and serotonergic-system activities, highlighting the neuro-functional effects associated with the ingestion of MPs and pollutants.

#### 3.3.2. Effects on the Gills

The gills are one of the entry routes for microplastics into the organism [66]. During breathing, MPs can passively enter the gill chambers of fish with the water flow and may adhere to the gill filaments. MPs were found in the gills of several fish species [26,33,67]. Fibers, fragments, and pellets are the most reported shapes isolated from gill fish. Fibers adhere easily to the gill filaments due to their physical properties [66]. 

Barboza et al. [33] found that the gills of the specimens that ingested MPs showed higher LPO levels and a higher index of lipid peroxidation damage than the specimens that were negative to MPs’ ingestion. Gill lipid peroxidation damage can lead to adverse effects, including compromise of respiration and biotransformation of xenobiotics [68]. Histologically, no tissue damage was reported at the gill level [44] (see Table 1). 

#### 3.3.3. Effects on the Muscle

MPs were found in the muscle tissue of fish species [39]. Considering that fish muscle is the part that is consumed by humans and that the presence of microplastics accumulated in this part of the body could be dangerous for human health, Ferrante et al. [69] highlighted the presence of MPs < 3μm in some seafood specimens (*Sparus aurata, Solea solea,* and *Mytilus galloprovincialis*) from the south coast of the Mediterranean Sea.

Barboza et al. [33] investigated the MPs’ contamination and the biomarkers effect as the total cholinesterase (ChE) and lipid peroxidation in wild *Dicentrarchus labrax*, *Trachurus trachurus,* and *Scomber colias*. The results showed that no significant differences were found in the ChE activity in muscles between fish that ingested MPs and others that had not ingested MPs. Moreover, the authors found that specimens that ingested MPs showed higher lipid peroxidation levels in the dorsal muscle than fish that had not ingested MPs.

Lipid peroxidation in muscle may alter muscular and neuro-muscular functions, resulting in an energy deficit, problems in coordination movements, and a decrease in swimming performance [70]. Barboza et al. [33] found high lipid peroxidation in fish that ingested MPs (see Table 1).

#### 3.3.4. Effects on the Liver

The liver plays an important role in the detoxification processes of xenobiotics, and consequently, this tissue is often used as an indicator of the degree of damage induced by pollutants. In the laboratory, it was demonstrated that MPs can cross the intestinal barrier, travel through the bloodstream, and reach the liver [71]. The bioaccumulation of MPs in the liver was also demonstrated in naturally exposed animals, such as birds and fishes (*Gadus morhua, Serranus scriba, Engraulis encrasicolus, Sardina pilchardus*, and *Clupea harengus*), by chemical analysis (pyrolysis gas chromatography–mass spectrometry) and polarized light microscopy [39,41]. The MPs’ accumulation was mainly in the blood vessels and the surrounding area [43]. Until now, no specific histopathological lesions at the hepatic level were found [39].

The toxic effects of MPs on *Serranus scriba* liver were recently evaluated by Zitouni et al. [43]. The catalase and glutathione S-transferase activities and the malondialdehyde content were monitored in sites with different anthropogenic impacts, as well as the acetylcholinesterase activity (nervous system enzyme) and metallothionein content. Significant site-dependent cytotoxicity in relation to MPs’ ingestion was pointed out [43]. Moreover, changes in malondialdehyde content were also observed, as well as the presence of reactive oxygen species (ROS) expressed by the altered levels of catalase and glutathione-S-transferase activities and in the content of metallothioneins (MTs).

The possible effect of the ingestion of MPs on the instauration of oxidative stress in the liver of *Seriola dumerili* was recently studied by Solomando et al. [48]. Catalase, superoxide dismutase, and glutathione S transferase showed increased activities in fish with a higher load of MPs with respect to those specimens that presented fewer MPs; a linear relationship between the number of MPs and the catalase and superoxide dismutase activities was also reported. The EROD activity and malondialdehyde levels were similar in both groups.

The GST enzyme was widely used as a biomarker of the detoxification system [36]. Alomar et al. [50] reported a significant increase in GST activity also in a *Mullus surmuletus* liver which had ingested MPs (see Table 1).

#### 3.3.5. Effects on the Digestive System (Gut)

In fish, the inflammatory activation is upregulated by cytokines, which are used as markers of inflammation [72]. The effect of MPs’ ingestion on the molecular signaling underlying intestinal inflammation was evaluated in two important commercial fish species, *Mullus barbatus* and *Merluccius merluccius* [51]. It was observed by the same authors that MPs’ abundance was highly correlated to cytokines (i.e., interleukin-1β, interleukin 10, and interferon). Moreover, CAT and SOD transcript levels suggested ROS generation and infiltration of immune cells in the gut [51].

As concerns marine invertebrates, in *Holoturia tubulosa*, the effects of MPs, considering a battery of biomarkers of oxidative stress (catalase, superoxide dismutase, glutathione reductase, and glutathione S-transferase and reduces glutathione) and neurotoxicity (acetylcholinesterase), were evaluated [49]. It was observed that the intestine of *H. tubulosa* from the most polluted areas showed higher CAT, SOD, glutathione reductase (GRd), and glutathione S-transferase (GST) activities and reduced glutathione (GSH) levels than those from the control area. A positive correlation between the presence of MPs in the intestine and CAT, GST, and GSH was found. Moreover, the levels of malondialdehyde presented similar values in all areas, thus indicating that the induction of antioxidant defense mechanisms had maintained the homeostasis of the organism and avoided oxidative damage. The acetylcholinesterase activity presented similar values in all areas, indicating the absence of neurotoxicity.

The effect of MPs’ ingestion on the gut microbiota was recently evaluated in the loggerhead sea turtle (*Caretta caretta*) [54]. During the transit through the digestive system, MPs can interact with the residing microbial community, with possible negative consequences on the host’s health. Putative pathogens were found in fecal samples that were characterized by high amounts of MPs. The hypothesis is that MPs can act as a carrier for pathogens (bacteria) in marine organisms. Different phylotypes associated with high levels of MPs were also identified (i.e., *Cetobacterium somerae* and other taxa), potentially responding to plastic-associated chemicals (see Table 1).

#### 3.3.6. Effects on the Endocrine System

On their surface, MPs can adsorb many chemical contaminants, such as antibiotics, heavy metals, phthalates, dioxins, organochlorine contaminants (HCB, DDTs, and PCBs), bisphenol A (BPA), and persistent organic pollutants (POPs) [73,74]. As reported by de Sá et al. [75], MPs can act as a carrier of pollutants in the marine food web.

Almost all plastic products are carriers of endocrine-disrupting chemicals (EDCs) [76], a wide range of substances, including pharmaceuticals, dioxin, and dioxin-like compounds; polychlorinated biphenyls; DDT and other pesticides; and components of plastics, such as BPA and phthalates. EDCs interfere with the body’s endocrine system by altering the synthesis, secretion, transport, activity, and elimination of hormones. EDCs can alter fish reproduction at various organizational levels. The adverse effect of plastic ingestion on *Trachurus trachurus* health was evaluated by using the liver expression of vitellogenin (VTG) as a biomarker for endocrine disruption [57]. The expression of VTG was observed in the liver of male specimens. The authors considered VTG expression as an indicator of xenoestrogen exposure that could be caused by the ingested plastics or by EDCs present in seawater whose effects can be exacerbated by plastics (see Table 1).

#### 3.3.7. Effects on Metabolism 

One of the organs most affected by problems related to the metabolic alterations linked to the MPs’ ingestion is the liver Chae et al. [77]. Some authors demonstrated that MPs’ exposure causes metabolic disorders in experimental conditions [68,77]. Zitouni et al. [43] performed an innovative metabolomic analysis on 36 metabolites in *Serranus scriba* liver. The metabolites investigated, mainly involved in energy, amino acid, and osmolyte metabolism, were significantly affected by the presence of MPs. On the contrary, Mancuso et al. [46], analyzing the amino acids and fatty acids levels (eye and liver) in relation to the presence of MPs in the GIT of *Scyliorhinus canicula*, did not find any effect or correlation between MPs’ abundance and amino acids and fatty acid (see Table 1).

#### 3.3.8. Genotoxicity

Zitouni et al. [43] estimated the potential genotoxicity associated with MPs uptake in the liver of *Serranus scriba*. The mutagenic potential of MPs was evaluated by the micronucleus test. Authors reported significant site-dependent genotoxicity by changes in the amount of micronucleus (see Table 1).

#### 3.3.9. Condition and Health Indicators

Condition factor and organo-somatic indices are general indicators of fish health status. The relative condition factor (Kn) and Fulton’s condition factor (CF) are most used and are based on the assumption that heavier fish of a given length are in better condition [78,79,80,81]. They are used in fishery science to assess the health status at a stock level, as well as to evaluate the impact of parasites on marine organisms [82,83,84]. Monitoring the well-being of fish could be very important in assessing the effects of MPs’ ingestion on marine organisms. Measuring fish conditions may provide a simple way to evaluate the quality of environmental conditions and their relationship with microplastic ingestion.

The influence of ingested MPs on the well-being of marine organisms was evaluated in some studies, most of them regarding teleosts (*Mullus barbatus, Gadus morhua, Limanda limanda, Platichthys flesus Clupea harengus, Scomber scombrus, Dicentrarchus labrax, Scomber colias, Boops boops, Sardina pilchardus*, and *Engraulis encrasicolus*), as well as elasmobranchs (*Scylhiorinus canicula*) and crustacean decapods (*Aristeus antennatus*) (Table 1). Some studies reported no direct effect of MPs’ ingestion on health status through using Fulton’s condition [33,44,45,52,53,56,59] or Kn [46,47]. Sbrana et al. [47] reported that individuals (*Boops boops*) in worse physical condition ingested MPs more frequently than individuals in better conditions (high values of Kn).

The same authors reported that the ingestions of MPs by fish living in worse environmental conditions (low values of Kn) could reflect the level of environmental contamination. Similarly, Compa et al. [55] reported, in *Sardina pilchardus*, higher levels of ingestion in fish with the lower condition, while the highest condition factor was found in fish with low levels of ingestion.

Carreras-Colom et al. [56] did not find negative effects; they only found a negative correlation between the fiber load and the gonadosomatic and hepatosomatic index (GSI) in *Aristeus antennatus* caught along the norther coast of Spain.

Rodríguez-Romeu et al. [44] evaluated the potential effect of anthropogenic (natural and synthetic) fiber ingestion in *Mullus barbatus*, using health-status indicators (Table 1). Hepatosomatic and gonadosomatic indices, Fulton’s body condition factor, and stomach-fullness index (as a measure of feeding intensity) showed no differences related to MPs ingestion levels (see Table 1).

## 4. Discussion

Although research on the effects caused by plastic ingestion has rapidly increased in the last decade, most of these studies were carried out in experimental conditions. These studies investigated the effects on feeding, reproduction, growth, development, and lifespan, whereas few articles reported the effects of MPs in wild marine species. This is mainly due to the difficulties in controlling or monitoring multiple environmental variables, such as feeding history [85].

The laboratory experiments allow for the assessment of various acute and chronic effects, even if they do not reproduce natural conditions. Most the experimental studies expose organisms (mainly zebrafish and medaka) to one type of polymer of a specific size (generally nanoplastics) and shape, but in the natural environment, organisms are exposed to a mixture of polymers with different sizes and shapes. Moreover, the doses used in experimental conditions greatly exceed the concentration found in naturally exposed marine organisms. It is important to underline that marine MPs can carry toxic chemicals on their surface. These toxins may cause further adverse effects on wild organisms.

Recently, some studies underlined the presence of natural and semisynthetic microfibers in wild marine organisms [86,87,88,89]. Although natural microfibers degrade faster than synthetic polymers, these microfibers can persist in the marine environment for a lot of time in relation to their nature and environmental factors [86]. Moreover, the degradation of natural and semisynthetic fibers causes the release of toxics adsorbed (i.e., textile dyes) to the surface into the environment [90,91]. In conclusion, the authors suggest paying more attention to the detection of dyes in natural fibers since these are indicators of anthropogenic processing and could cause biological damage.

The knowledge of adverse effects caused by direct ingestion of microplastics in wild animals is still limited; more efforts are needed to fully understand the role of MPs in marine ecosystems and their adverse effects on wild organisms.

Plastic pollution is a threat affecting marine ecosystems globally. The amount of plastic in the natural environment is continuously increasing. To contrast this phenomenon, multilevel mitigation strategies are being addressed for the reduction of plastic waste, the improvement of waste management, and the recovery of polluted areas. Nevertheless, huge efforts are needed to drastically reduce plastic emissions in aquatic ecosystems in an acceptable temporal range. The entire global plastics economy should be transformed.

## Figures and Tables

**Table 1:** Summary of effects caused by microplastic ingestion in wild marine biota.

Area	Species	N Specimens	% Specimens with Ingested MPs	Biomarker	Effect	Reference
Northeast Atlantic Ocean	Seabass, *Dicentrarchus labrax*, Horse mackerel, *Trachurus trachurus*, Atlantic chub mackerel, *Scomber colias*	150	49	Fulton’s condition factor (CF)	No effect	[33]
				Brain acetylcholinesterase (AChE) activity	Altered activity (↑)	
				Muscle total cholinesterases (ChE) activity	No effect	
				Brain, muscle, and gills lipid peroxidation (LPO)	Altered levels (↑)	
Strait of Sicily	Painted comber, *Serranus scriba*	120	22–43	Catalase (CAT) activity	Altered level (↑)	[43]
				Glutathione S-transferase (GST) activity	Altered level (↑)	
				Malondialdehyde (MDA) content	Altered level (↑)	
				Acetylcholinesterase (AChE) activity	Altered level (↓)	
NW Mediterranean Sea	Red mullet, *Mullus barbatus*	118	59	Gonado-somatic index (GSI)	No effect	[44]
				Hepato-somatic index (HSI)	No effect	
				Stomach-fullness index (FULL)	No effect	
				Fulton’s body condition factor (CF)	No effect	
				Gonad, liver, spleen, kidney, stomach, and gill histology	No tissue damage	
North Sea	Cod, *Gadus morhua* Flounder, *Paralichthys dentatus* Sawbill duck, *Mergus merganser* Common guillemot, *Uria aalge*	13	61.5	Organ’s histology	No tissue damage	[39]
South of Sicily	Small-spotted catshark, *Scylhiorinus canicula*	50	80	Gonado-somatic index (GSI)	No effect	[45]
				Hepato-somatic index (HSI)	No effect	
				Spleno-somatic index (SSI)	No effect	
				Fulton’s body condition factor (CF)	No effect	
				Immune-related gene expression	No effect	
South of Sicily	Small-spotted catshark, *Scylhiorinus canicula*	61	80.3	Relative condition factor (Kn)	No effect	[46]
				Ammino acids and fatty acids profiles	No effect	
Southern Tyrrhenian Sea	Bogue, *Boops boops*	65	Na	Relative condition factor (Kn)	No effect	[8]
Tyrrhenian and Ligurian Seas	Bogue, *Boops boops*	379	56	Relative condition factor (Kn)	No effect	[47]
Western Mediterranean Sea	Amberjack, *Seriola dumerili*	52	98	Superoxide dismutase (SOD) activity	Altered level (↑)	[48]
				Catalase (CAT) activity	Altered level (↑)	
				Glutathione S-transferase (GST) activity	Altered level (↑)	
				Ethoxyresorufin-O-deethylase (EROD) activity	No effect	
				Malondialdehyde (MDA)	No effect	
Western Mediterranean Sea	Sea cucumber, *Holothuria tubulosa*	30	83.3	Superoxide dismutase (SOD) activity	Altered level (↑)	[49]
				Catalase (CAT) activity	Altered level (↑)	
				Glutathione reductase (GRd) activity	Altered level (↑)	
				Glutathione S-transferase (GST) activity	Altered level (↑)	
				Acetylcholinesterase (AChE) activity	No effect	
				Malondialdehyde (MDA)	No effect	
Western Mediterranean Sea	Striped mullet, *Mullus surmuletus*	417	27.3	Superoxide dismutase (SOD) activity	No effect	[50]
				Catalase (CAT) activity	No effect	
				Glutathione S-transferase (GST) activity	Altered level (↑)	
				Malondialdehyde (MDA)	No effect	
Central Adriatic Sea	Red mullet, *Mullus barbatus*	16	na	Interleukin-1beta (IL-1β)	Altered level (↑)	[51]
				Interleukin IL-8	Altered level (↑)	
				Interleukin IL-10	Altered level (↑)	
				Interferon (IFN)	Altered level (↑)	
				Catalase (CAT) activity	Altered level (↑)	
				Superoxide dismutase (SOD) activity	Altered level (↑)	
Central Adriatic Sea	European hake, *Merluccius merluccius*	16	na	Interleukin-1beta (IL-1β)	Altered level (↑)	[51]
				Interleukin IL-8	No effect	
				Interleukin IL-10	Altered level (↑)	
				Interferon (IFN)	Altered level (↑)	
				Catalase (CAT) activity	Altered level (↑)	
				Superoxide dismutase (SOD) activity	Altered level (↑)	
North and Baltic Seas	Atlantic cod, *Gadus morhua,* Dab, *Limanda limanda,* European flounder, *Platichthys flesus* Atlantic herring, *Clupea harengus* Atlantic mackerel, *Scomber scombrus*	290	5.5	Fulton’s body condition factor (CF)	No effect	[52]
Greenland Sea	Bigeye sculpin, *Triglops nybelini* Polar cod, *Boreogadus saida*	156	18–34	Fulton’s body condition factor (CF)	No effect	[53]
Adriatic Sea	Loggerhead turtle, *Caretta caretta*	45	98	V3/V4 hypervariable region of 16s rRNA	Operational taxonomic units (OTUs) variation	[54]
Western Mediterranean Sea	European sardine, *Sardina pilchardus* Anchovy *Engraulis, encrasicolus*	210	14.3–15.2	Fulton’s body condition factor (CF)	Altered level (↓)	[55]
Northwestern Mediterranean Sea	Deep-water shrimp, *Aristeus antennatus*	148	39.2	Condition indices (K, hepatosomatic index)	No effect	[56]
Central Mediterranean Sea	Atlantic horse mackerel, *Trachurus trachurus*	92	90.6	Vitellogenin (VTG)	Altered level (↑)	[57]
Western Mediterranean Sea	Anchovy, *Engraulis encrasicolus* Striped mullet, *Mullus surmuletus* Bogue, *Boops boops*	34 44 51	-	Fulton’s body condition factor (CF)	No effect	[58]
				Catalase (CAT) activity	Altered level (↑) in *E. encrasicolus*	
				Superoxide dismutase (SOD) activity	Altered level (↑) in *E. encrasicolus*	
				Glutathione S-transferase (GST) activity	Altered level (↑) in *M. surmuletus*	
				Acetylcholinesterase (AChE) activity	No effect	
				Malondialdehyde (MDA)	No effect	
Western Mediterranean Sea	Bogue, *Boops boops*	102	46	Fulton’s Body condition factor (CF)	No effect	[59]
Central and Northwestern Mediterranean Sea	Fin whales, *Balenoptera physalus*	36 34		CYP1A in skin biopsy	Altered level (↑)	[60]
				CYP2B in skin biopsy	Altered level (↓)	
				lipid peroxidation (LPO) in skin biopsy	Altered level (↑)	
Northwestern Mediterranean Sea	Deep-water shrimp, *Aristeus antennatus*	201	75.1	Relative condition factor (Kn)	No effect	[61]
				Gonado-somatic index (GSI)	Altered level (↓)	
				Hepato-somatic index (HSI)	Altered level (↑)	

N: number; (↑): increase; (↓): decrease.

**Table 2:** Gender ratio among scientists working on the effects of MPs on marine biota.

Author Role	F	M	% Females	Reference
Last	6	4	60	[33]
First	6	5	55	[43]
Last	3	2	60	[44]
First and last	3	2	60	[39]
First and last	5	4	56	[45]
First and last	7	6	54	[46]
First and last	8	3	73	[8]
First	3	7	30	[47]
First	3	3	50	[48]
First	5	4	56	[49]
First and last	4	2	67	[50]
-	2	4	33	[51]
-	1	6	14	[52]
First and last	5	4	56	[53]
First	6	4	60	[54]
First and last	5	0	100	[55]
First and last	5	2	71	[56]
Last	6	1	86	[58]
Last	5	2	71	[59]
First and last	5	5	50	[57]
First and last	5	1	83	[61]
First and last	8	7	53	[60]

F: female; M: male.

## Data Availability

The data presented in this study are available on request from the corresponding author.
